# Evaluation of radiofrequency identification tag accuracy using bronchoscopy with fluoroscopy and virtual navigation guidance before segmentectomy

**DOI:** 10.1007/s00464-024-11110-4

**Published:** 2024-08-01

**Authors:** Masamichi Komatsu, Kentaro Miura, Miwa Yamanaka, Yusuke Suzuki, Taisuke Araki, Norihiko Goto, Jumpei Akahane, Kei Sonehara, Shunichiro Matsuoka, Takashi Eguchi, Kazutoshi Hamanaka, Kimihiro Shimizu, Masanori Yasuo, Masayuki Hanaoka

**Affiliations:** 1 First Department of Internal Medicine, Shinshu University School of Medicine, Matsumoto, Japan; 2 Division of General Thoracic Surgery, Department of Surgery, Shinshu University School of Medicine, Matsumoto, Japan; 3 Department of Clinical Laboratory Sciences, Shinshu University School of Health Science, Matsumoto, Japan

**Keywords:** Bronchoscopy, Lung cancer, Radiofrequency identification, Segmentectomy, Sublobar resection, Virtual bronchoscopic navigation

## Abstract

**Background:**

The use of sublobar resection has increased with advances in imaging technologies. However, it is difficult for thoracic surgeons to identify small lung tumours intraoperatively. Radiofrequency identification (RFID) lung-marking systems are useful for overcoming this difficulty; however, accurate placement is essential for maximum effectiveness.

**Methods:**

We retrospectively reviewed patients who underwent RFID tag placement via fluoroscopic bronchoscopy under virtual bronchoscopic navigation (VBN) guidance before our institution’s sublobar resection of lung lesions. Thirty-one patients with 31 lung lesions underwent RFID lung-marking with fluoroscopic bronchoscopy under VBN guidance. Results: Of the 31 procedures, 26 tags were placed within 10 mm of the target site, 2 were placed more than 10 mm away from the target site, and 3 were placed in a different area from the target bronchus. No clinical complications were associated with RFID tag placement, such as pneumothorax or bleeding. The contribution of the RFID lung-marking system to surgery was high, particularly when the RFID tag was placed at the target site and tumour was located in the intermediate hilar zone.

**Conclusions:**

An RFID tag can be placed near the target site using fluoroscopic bronchoscopy in combination with VBN guidance. RFID tag placement under fluoroscopic bronchoscopy with VBN guidance is useful for certain segmentectomies.

**Supplementary Information:**

The online version contains supplementary material available at 10.1007/s00464-024-11110-4.

Lobectomy has been the standard evidence-based surgical treatment for early-stage lung cancer for many years [1]. However, in recent years, randomised controlled studies showed that segmentectomy for selected patients with peripherally located early-stage lung cancer had equivalent outcomes compared to lobectomy [2, 3]. In addition, the use of sublobar resection has been increasing in elderly patients with small nodules on computed tomography (CT), such as those with metastatic lung cancer and/or lung cancer [4, 5]. However, lung segmentectomy is a technically challenging procedure for thoracic surgeons [6]. It is difficult to identify and resect tumours with sufficient margins during surgery, especially nonpalpable nodules. It has been reported that conversion to thoracotomy is required in 54% of cases when the ground-glass nodule (GGN) is located deep in the visceral pleura due to the difficulty localising pulmonary nodules [7].

To overcome this difficulty, various marking systems have been developed to identify small pulmonary nodules. CT-guided percutaneous puncture, especially with hook-wire placement at the lung surface near the target lesion, is a lung-marking method used worldwide [8, 9]. However, the high frequency of complications associated with hook-wire placement, such as pneumothorax or bleeding, is a problem [10, 11]. Air embolism, which occurs in 0.06% of the cases, is usually a fatal outcome [12]. To avoid these complications, a lung-marking system was developed using bronchoscopy. Sato et al. developed virtual-assisted lung mapping (VAL-MAP) using multiple-dye markings of the lung surface via bronchoscopy [13]. This multicentre study demonstrated the safety and efficacy of the VAL-MAP method in lung resection [14].

Radiofrequency identification (RFID) lung-marking system is a newly developed technique for detecting tumour localisation (SuReFInD; Hogy Medical Co., Ltd., Tokyo, Japan) [15, 16]. This system consisted of (a) a bronchoscopic delivery device, (b) a micro-RFID tag with a coil anchor as a marker, and (c) a detection probe with a signal processing device (Fig. 1). Before surgery, an RFID tag was placed adjacent to the tumour via bronchoscopy. Thoracic surgeons can easily identify RFID tag locations using signal sounds during surgery. This system is helpful in 93% of cases evaluated by thoracic surgeons for sublobar resection when an RFID tag is placed under a combination of cone-beam CT and bronchoscopy [17].

**Fig. 1:**
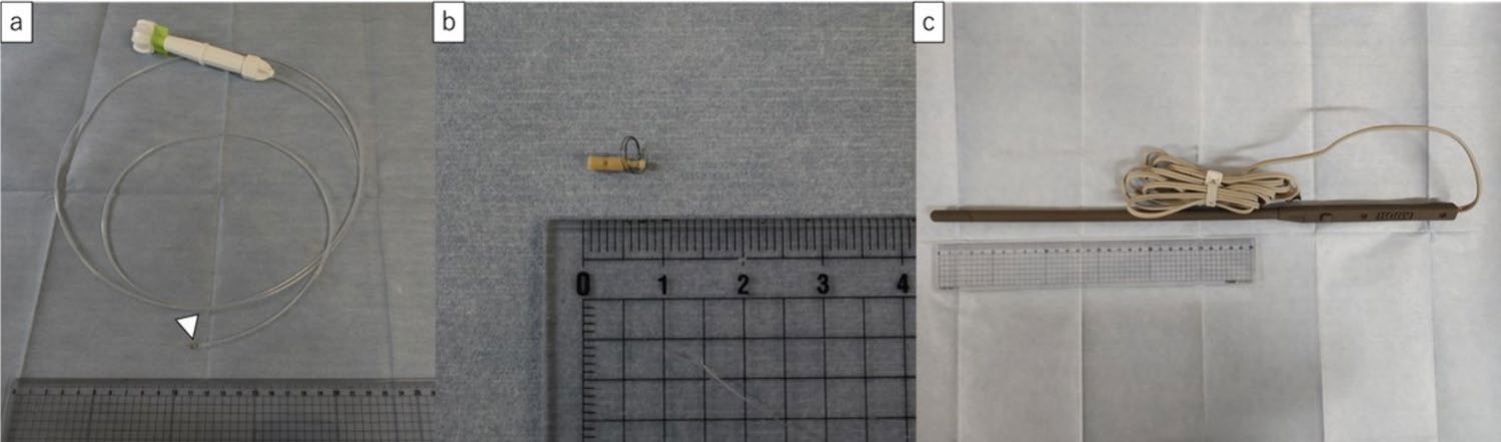
Composition of the RFID lung-marking system. **a** A bronchoscopic delivery device, **b** a micro-RFID tag with a coil anchor as a marker. A micro-RFID tag is stored at the tip of the bronchoscopic delivery device (arrowhead). **c** A detection probe during surgery with a signal processing device. *RFID* radiofrequency identification

The most essential aspect of the RFID lung-marking system is the accurate placement of the RFID tag at the target site. To achieve this, RFID tag placement is generally performed under cone-beam CT to confirm the placement site [15–20]. However, institutions where cone-beam CT can be used are limited, and a new strategy is needed to spread the RFID lung-marking system widely.

This study aimed to evaluate the accuracy of RFID tag placement using flexible bronchoscopy with fluoroscopy and virtual bronchoscopic navigation (VBN) guidance before surgical resection. Moreover, we evaluated the efficacy and safety of the RFID lung-marking system followed by segmentectomy in patients with small lung nodules.

## Methods

### Study subjects

The Institutional Review Board of Shinshu University approved this retrospective single-centre study (approval number: 5755). The study was conducted in accordance with the principles of the Declaration of Helsinki, and because of the retrospective study design, the Institutional Review Board waived the need for informed consent.

### Study design

This study evaluated the accuracy of RFID tag placement using flexible bronchoscopy with fluoroscopy and virtual bronchoscopic navigation (VBN) guidance before surgical resection. We further evaluated the efficacy and safety of the RFID lung-marking system followed by segmentectomy in patients with small lung nodules. We reviewed the medical records of 34 consecutive patients who underwent segmentectomy using the RFID lung-marking system between July 2020 and December 2023. Thoracic surgeons decided to use RFID lung-marking systems. RFID lung-marking systems were used for segmentectomy to resect pulmonary nodules and/or lesions that were anticipated to be hardly palpable during the operation and/or in which the resection margin needed to be carefully selected regardless of the palpability of the lesion.

### Procedures

#### Setting the target RFID tag placement site

**Fig. 2:**
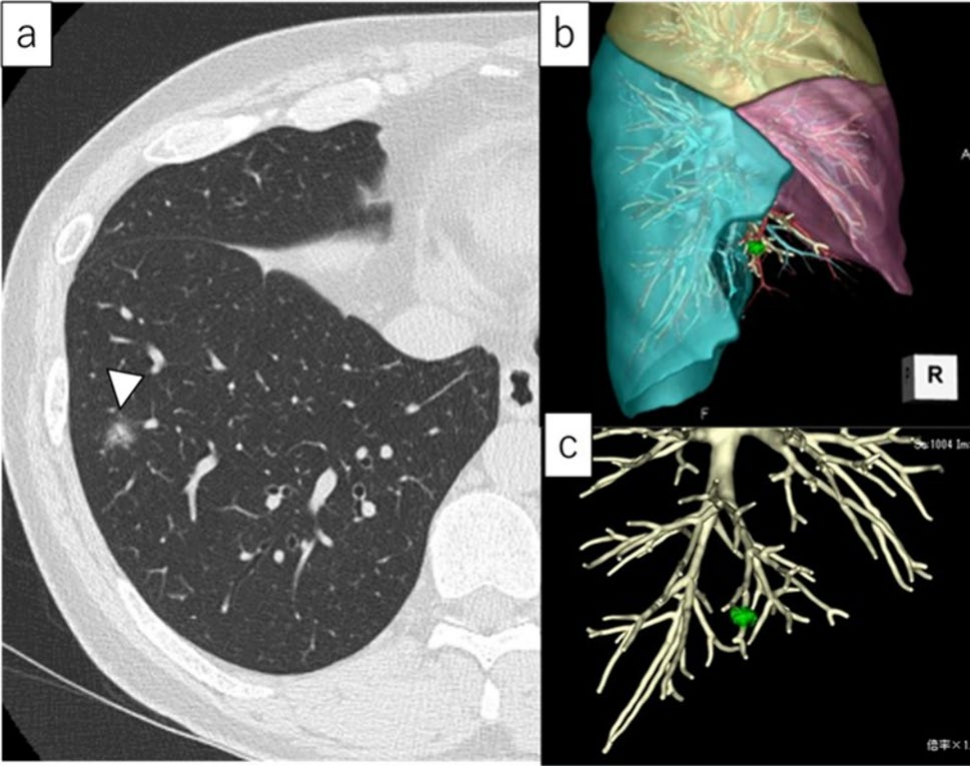
Preoperative planning and simulation of RFID tag placement. A 47-year-old male presented with an abnormal shadow. **a** CT scan revealed an 11 mm pure GGN at the right S8a (arrowhead). For diagnosis and treatment, right S7 + 8 resection was planned. **b**, **c** Preoperative planning and simulation of sublobar resection using three-dimensional CT images and volume-rendering reconstruction software. After discussions with the thoracic surgeon and bronchoscopist, the target site for RFID tag placement was decided to be on the dorsal side of the tumour. *CT,*computed tomography, *GGN* ground-glass nodule, *RFID* radiofrequency identification

Preoperative planning and simulation of the segmentectomy were performed by thoracic surgeons using three-dimensional CT images and volume-rendering reconstruction software (REVORAS, Ziosoft, Inc. Tokyo, Japan) [21]. Next, the thoracic surgeons and bronchoscopist determined the target site for RFID tag placement based on preoperative simulation. Depending on the case, it was decided whether the target placement site was near the tumour to secure surgical margins or to identify the target bronchi during segmentectomy. Discussions with the thoracic surgeons and bronchoscopists determined the number of tags to be placed. Figure 2 shows the preoperative planning and simulation of RFID tag placement.

The target tumour’s location, longest diameter, and appearance on CT (pure GGN, part-solid nodule, solid nodule, or other) were assessed. The distance from the pleura to the target tumour and from the hilum on CT (central, intermediate, or peripheral third zone), as classified by Yasuo et al. [22], were also evaluated.

#### Bronchoscopic procedure for RFID tag marking

A chest CT image with a 0.63–1.00 mm width slice was acquired before bronchoscopy. Data from the chest CT images were inputted into the VBN system (LungPoint; Bronchus Technologies, Inc., CA, USA, from July 2020 to March 2022, and DirectPath; Cybernet Systems, Tokyo, Japan, from April 2022 to December 2023). Using a VBN system, a virtual bronchoscopic pathway indicating the bronchial route to the target site was created. A VBN image is shown in Video 1.

**Fig. 3:**
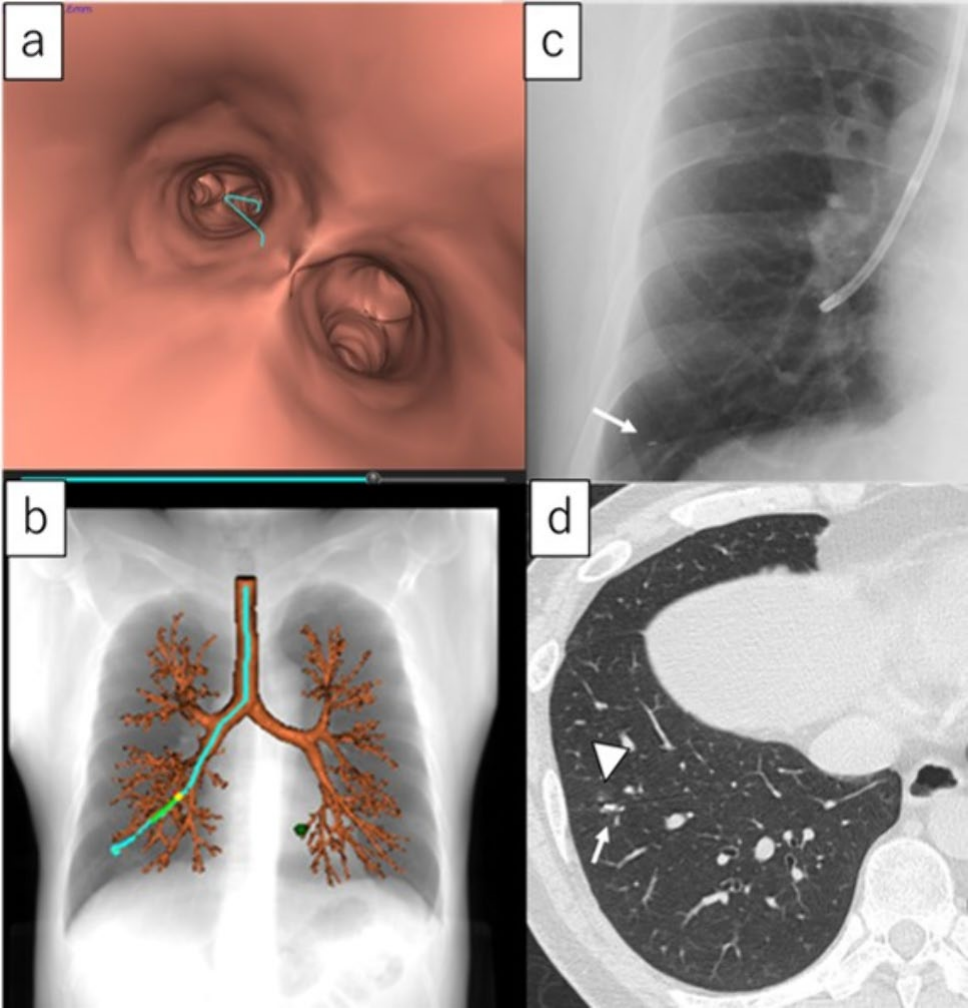
Virtual bronchoscopic image and bronchoscopy for RFID tag placement. The same patients presented in Fig. 2. **a**, **b** A virtual bronchoscopic pathway for the target site is created using a VBN system. **c** An RFID tag (arrow) is placed at the target site using a thin bronchoscope with fluoroscopy in combination with VBN guidance. **d** Chest CT image after bronchoscopy reveals the relationship between the actual RFID tag (arrow) location and target lesion. The actual RFID tag was located on the dorsal side of the tumour (arrowhead), which was judged to be ‘complete.’ Three days after RFID tag placement, the patient underwent robot-assisted thoracoscopic surgery. *CT* computed tomography, *RFID* radiofrequency identification, *VBN* virtual bronchoscopic navigation

Bronchoscopic procedures were performed under local anaesthesia with lidocaine and conscious sedation with intramuscular pethidine or intravenous combined midazolam and fentanyl. Bronchoscopy was performed using a 4.2 mm thin bronchoscope (BF-P290; Olympus, Tokyo, Japan). The RFID tag information is then registered in the application. After advancing the thin bronchoscope into the target bronchus as far as possible under VBN guidance, a 1.8 mm diameter bronchoscopic delivery device was advanced toward the target site via the working channel. The delivery device was advanced with reference to the virtual fluoroscopic image under VBN. The tip of the delivery device was positioned near the target site while the distances to the diaphragm, heart, and ribs were checked using fluoroscopy. Then, the RFID tag placed inside the delivery device was ejected. Fluoroscopy confirmed that the RFID tag had been positioned near the target site. Figure 3 shows the virtual bronchoscopic image and bronchoscopy for RFID tag placement. The actual bronchoscopic procedures for RFID tag placement are shown in Video 2. The duration of the procedure was determined by the insertion of the bronchoscope into the trachea for removal. The bronchus level was reached with a bronchoscope (subsegmental bronchi were regarded as third-generation bronchi, and bronchial generation was calculated by adding the number of branches, as previously reported by Oki et al. [23]) was also recorded.

#### Confirming the RFID tag placement site

**Fig. 4:**
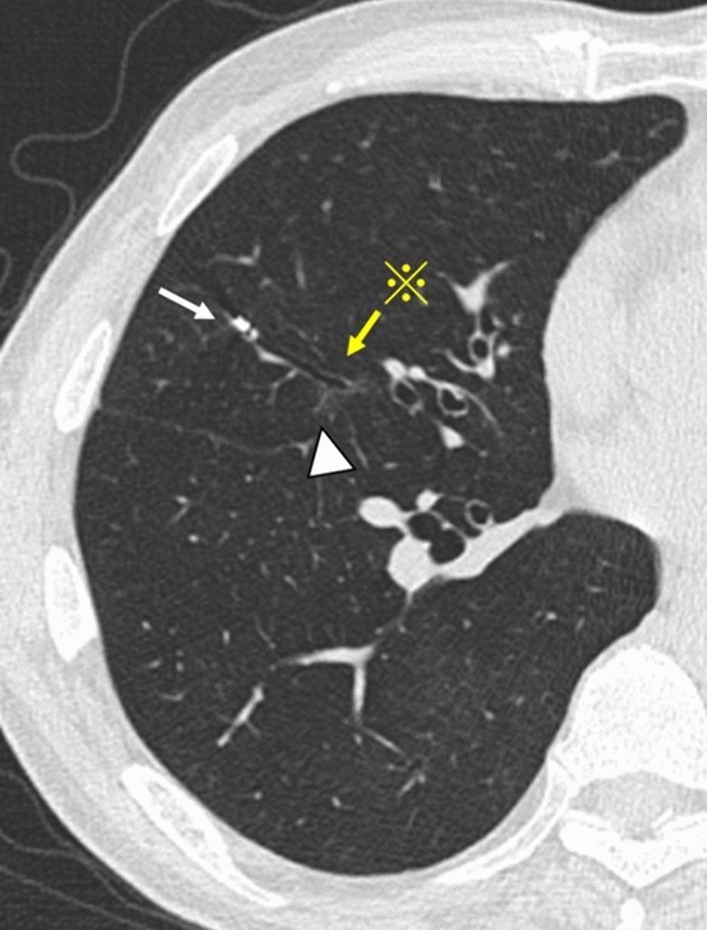
Deviation between the targe placement site and the actual placement site. The upper margin and ventral side of the tumour (arrowhead) were set as the target placement sites (asterisk, yellow arrow). The actual RFID tag placement site is distal to the target site (white arrow). The distance from the RFID tag to the target site was 20.8 mm, which was judged to be ‘incomplete.’ *RFID* radiofrequency identification, *CT* computed tomography, *GGN* ground-glass nodule, *RFID* radiofrequency identification, *VBN* virtual bronchoscopic navigation.

The patient underwent chest CT following bronchoscopy. Thoracic surgeons and bronchoscopists checked the actual RFID tag placement locations using CT. Thoracic surgeons redetermined the surgical planning and approach. We defined the deviation between the target and actual placement sites as follows: (1) complete, within 10 mm from the target placement site; (2) incomplete, more than 10 mm from the target placement site; and (3) failure, placement in a different bronchus. A representative case is shown in Fig. 4 and Video 3.

#### Surgical procedures

The patient underwent segmentectomy within 72 h of RFID tag placement. The RFID tag was identified intraoperatively using a handheld sterile detection probe. Postoperatively, radiographs confirmed the presence of an RFID tag inside the surgical specimen (Figure S1 in the Supporting Information). The extent of the contribution to surgery made by the RFID lung-marking system was evaluated by the surgeons as reported previously [14, 17] as follows: (1) necessary, the same level of surgical precision, impossible without the RFID marking system; (2) useful, the RFID marking system enabled the confident performance of the operation; and (3) unnecessary, the same operation was possible without RFID.

## Results

### Patients and target lesion characteristics

During the study period, 327 patients underwent segmentectomy at our institution. Of these, 34 (10.4%) underwent surgery using the RFID lung-marking system. Among the 34 patients, three patients underwent bronchoscopy with cone-beam CT. The remaining 31 patients underwent fluoroscopic bronchoscopy. Combination use of VBN was performed in all cases during bronchoscopy. The subjects of this study were 31 patients who underwent bronchoscopy with fluoroscopy and VBN guidance.

In this study, an RFID tag was placed on each lesion. Thus, 31 patients with 31 lesions were marked with 31 RFID tags (Table 1). The median size of the target lesion and the median distance from the pleura were 10.8 mm and 21.4 mm, respectively.

**Table 1:** Characteristics of the patients and the target tumour

Patient characteristics	N = 31
Age, median (IQR), yr	68.6 (61.9–74.3)
Sex	
Male	20 (64.5)
Female	11 (35.5)
Tumour characteristics	N = 31
Tumour location	
Right upper lobe	5 (16.1)
Right middle lobe	1 (3.1)
Right lower lobe	10 (32.3)
Left upper lobe	9 (29.0)
Left lower lobe	6 (19.4.2)
Tumour size in the longest diameter, median (IQR), mm	10.8 (8.1–15.5)
Distance from the pleura, median (IQR), mm	21.4 (13.0–30.1)
Tumour location from the hilum	
Central	0 (0.0)
Intermediate	16 (51.6)
Peripheral	15 (48.4)
Appearance on CT	
Pure GGN	16 (51.6)
Part-solid nodule	8 (25.8)
Solid nodule	5 (16.1)
Others (cavity)	2 (6.5)

Data have been presented as median (IQR), or *N* (%)

*CT* computed tomography, *GGN* ground-glass nodule, *IQR* interquartile range

### The outcome of RFID tag placement via bronchoscopy with fluoroscopy and VBN

The RFID tag placement results are listed in Table 2. Twenty-five patients underwent bronchoscopic procedures with intramuscular pethidine and six with intravenous midazolam and fentanyl. The median duration of the procedure was 10.0 min. The distances from the RFID tag to the target site and target tumour on the CT were 2.7 mm and 5.1 mm, respectively. In 26 cases (83.9%), RFID tag placement was achieved within 10 mm of the target site, defined as complete RFID tag placement. Two cases were incompletely placed, and three were placed in different bronchi, resulting in failure. No clinical complications were associated with RFID tag placement, such as pneumothorax or bleeding. One case of RFID tag dislodgement from the bronchus due to coughing immediately after bronchoscopy, so the RFID tag was placed again.

**Table 2:** Bronchoscopic procedure and RFID tag placement

Sedation during bronchoscpy	
Pethidine	25 (80.7)
Midazolam combined with fentanyl	6 (19.4)
Procedure time, median (IQR), min	10.0 (7.3–12.0)
Bronchial generations, median (range)	5 (4–6)
Distance from the RFID tag	
to the target site, median (IQR), mm (N = 28)	2.7 (0.8–4.0)
to the target tumour, median (IQR), mm (N = 31)	5.1 (2.9–9.7)
RFID tag placement	
Complete	26 (83.9)
Incomplete	2 (6.5)
Failed	3 (9.7)
Safety	
Pneumothorax	0 (0.0)
Bleeding	0 (0.0)
RFID tag dislodged	1 (3.2)
RFID misplacement requiring bronchoscopic removal	1 (3.2)
Timing of surgery after bronchoscopy	
One day after	5 (16.1)
Two days after	14 (45.2)
Three days after	12 (38.7)

Data are presented as median (range or IQR) or *N* (%)

*IQR* interquartile range, *RFID* radiofrequency identification

### Outcome of resection and contribution of the RFID system to surgery

**Fig. 5:**
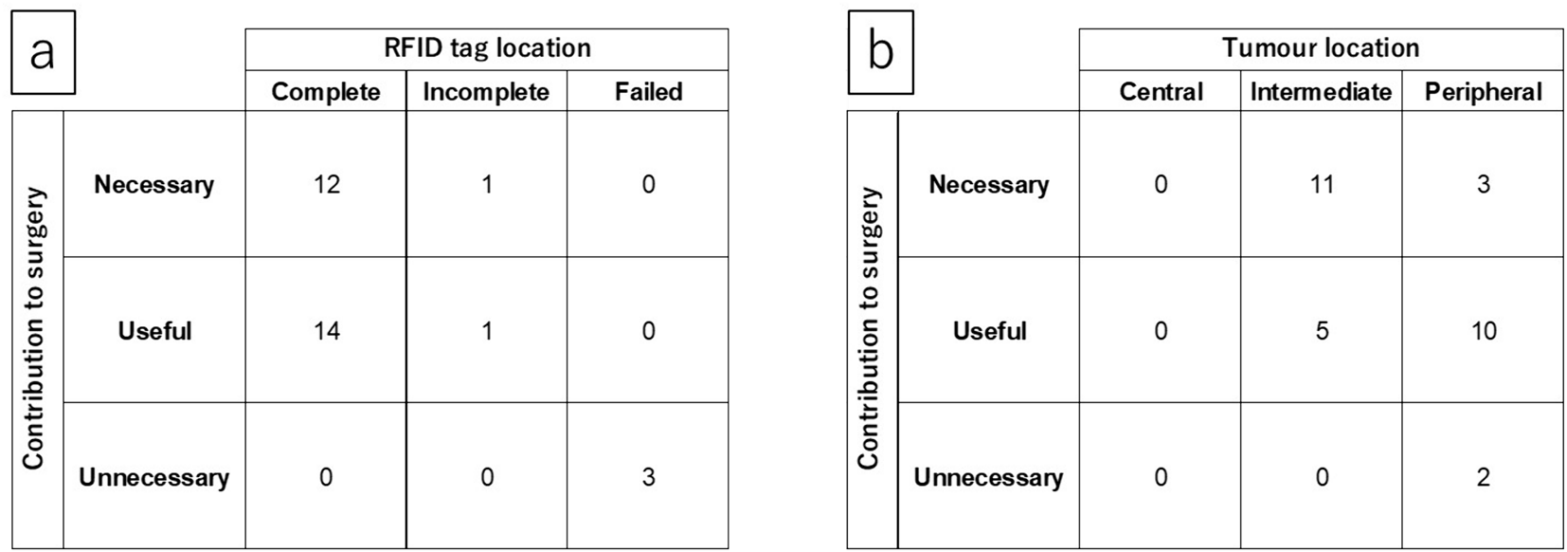
Correlation between tumour location and RFID markings’ contribution to the surgery. Relationship between **a** RFID tag locations, **b** tumour location and contribution to the surgery. *RFID* radiofrequency identification

The clinicopathological characteristics of the patients are presented in Table 3. Of the 31 patients, simple segmentectomy was performed in two cases, and complex segmentectomy in 29 patients. In one of the two cases judged to be incomplete, the surgical planning was changed after RFID tag placement, and in the remaining case, segmentectomy was performed without changing of surgical planning. In two of the three cases judged to be a failure, the surgical planning was changed after RFID tag placement, and segmentectomy was performed. In the remaining case, bronchoscopic removal of the RFID tag was performed, and the segmentectomy was performed without RFID lung marking. Pathological examination revealed 25 lung cancers: three pTis, 7 pTmi, 10 pT1a, 5 pT1b, and 6 metastatic tumours. Complete resection with sufficient margins was pathologically proven in all the lesions. Surgeons evaluated that the RFID lung-marking system was necessary for 41.9%, useful for 48.4%, and unnecessary for 9.7% of the patients. Figure 5a shows the relationship between the RFID tag placement site and its contribution to surgery. In many cases, when an RFID tag was placed near the target site, it was helpful for surgery, whereas when it was placed outside the target bronchus, it was insufficient. In addition, Fig. 5b shows the relationship between the tumour location and RFID markings’ contribution to the surgery. The RFID lung-marking system showed a particularly high contribution to tumours in the intermediate hilar zone. Table S1 demonstrates the relationship between the tumour appearance on CT and RFID markings’ contribution to the surgery. RFID lung-marking system showed a particularly high contribution to pure GGN on CT.

**Table 3:** Tumour characteristics and contribution of the RFID system to surgery

Operation types	
Simple segmentectomy	2 (6.5)
Complex segmentectomy	29 (93.5)
Surgical approach	
VATS	15 (48.4)
RATS	16 (51.6)
Pathological diagnosis	
Lung cancer	25 (80.7)
pTis	3
pT1mi	7
pT1a	10
pT1b	5
Metastatic lung cancer	6 (19.4)
Colorectal cancer	3
Uterine cancer	1
Pancreatic cancer	1
Seminoma	1
Contribution of the RFID tag to surgery	
Necessary	13 (41.9)
Useful	15 (48.4)
Unnecessary	3 (9.7)

Data have been presented as *N* (%)

*RATS* robot-assisted thoracoscopic surgery, *RFID* radiofrequency identification, *VATS* video-assisted thoracoscopic surgery

## Discussion

The RFID lung-marking system is a novel system that uses bronchoscopy. Using an RFID tag, it is possible to accurately confirm the tumour’s location using a real-time signal during surgery. Miyahara et al. [17] demonstrated the safety and effectiveness of the RFID lung-marking system during sublobar resection. However, the study was based on the RFID tag placement under cone-beam CT bronchoscopy. In fact, only a limited number of facilities allow cone-beam CT to be before sublobar resection.

Fluoroscopic bronchoscopy is a common and versatile examination technique. In addition, VBN improves the diagnostic yield of peripheral lesions involving the bronchi [24–26]. Therefore, we investigated whether using a VBN system to place an RFID tag at a precise location using fluoroscopic bronchoscopy was possible. This is the first comprehensive study on RFID tag placement using VBN in fluoroscopic bronchoscopy.

In this study, we demonstrated that an RFID tag could be placed in the bronchus within 10 mm of the target site in 83.9% of cases with fluoroscopic bronchoscopy using VBN guidance. In addition, the RFID marking system is useful for segmentectomy if an RFID tag is placed near the target site and the tumour is located in the intermediate hilar zone. No bronchoscopy-related complications, such as bleeding or pneumothorax, were observed.

This study demonstrated that the RFID lung-marking system highly contributes to surgeons for segmentectomy. Preeoperative simulation, consideration of the target placement site, and accurate placement are necessary to fully utilise the RFID lung-marking system. RFID lung-marking can confirm tumour locations, secure the surgical margins, and identify target bronchi. At our institution, the target placement site was determined through discussion between thoracic surgeons and bronchoscopists. We believe thoracic surgeons and bronchoscopists should thoroughly discuss and determine the appropriate target placement site. RFID lung-marking systems are often used to identify the target bronchus to sufficient surgical margin in our institution (data not shown). Therefore, placing the RFID tag in the target bronchus is more important than placing the RFID tag inside the tumour. In 28 cases (90.7%), excluding 3 cases, placement inside the target bronchus was achieved, which we believe contributed greatly to the success of this study. Furthermore, the RFID system can mark deep lesions in the visceral pleura, which is difficult with CT-guided hook-wire placement and the VAL-MAP system. This study found 16 lesions (51.6%) located in the intermediate hilar zone. This study also demonstrated that RFID lung marking is useful for the tumour located in the intermediate hilar zone.

Several points must be considered for accurate RFID tag placement. First, forwarding the bronchoscope and selecting as many bronchi as possible is essential, with the VBN as a reference. In addition, the virtual fluoroscopic image can be used as a reference to determine the position of the delivery device under fluoroscopy. In determining the placement site, it is important to measure the distance from the last selectable bronchus, as well as other markers that serve as references on fluoroscopic images (e.g., the ribs and heart) to the target site. A VBN system using virtual fluoroscopic images can be created from CT images.

The usefulness of fluoroscopic bronchoscopy in combination with electromagnetic navigation bronchoscopy (ENB), in terms of a high tumour resection rate with sufficient margins, has been reported [27]. We believe that the advantage of our method is that we can select as many bronchi as possible using a thin bronchoscope, whereas, in ENB, only a regular bronchoscope can be used. Furthermore, the combined use of VBN may shorten the bronchoscopic procedure time compared with ENB. Although it is difficult to make a simple comparison, it took 25.0 min when ENB was used [27] and 10.0 min when VBN was used. This is thought to be due to the ability to select a more distal bronchus using a thin bronchoscope with VBN as a reference. Reducing the bronchoscopy procedure time will lead to a reduction in radiation exposure.

Furthermore, CT scanning after RFID tag placement facilitates an understanding of the positional relationship between the RFID tag and the tumour. Our method allows thoracic surgeons to reconsider surgical planning and resect tumours with appropriate margins. In fact, surgical planning was changed after CT following RFID tag placement to successfully resect the tumour including RFID tags in three cases (one judged to be incomplete and two with failure).

There are also challenges to the RFID tag placement method using fluoroscopic bronchoscopy with VBN guidance. Although no pneumothorax or bleeding related to bronchoscopy was observed, there was one case of RFID tag dislodgement immediately after bronchoscopy. Dislodgement of the RFID tag is caused by placement in a bronchus with a diameter of 3.0 mm or more, so it is important to confirm the thickness of the bronchus at the target placement site. In other cases, there was a time lag of up to 72 h between RFID tag placement and surgery, but chest radiographs showed no migration of the RFID tag.

In addition, the RFID tag was misplaced at a bronchus different from the target placement site in three cases. In one case, the RFID tag was mislocated to a location significantly different from the target placement site and required removal via bronchoscopy. In all three cases where the RFID was placed in a different bronchus, the last bronchus navigated by the VBN could not be selected with a thin bronchoscope. It was placed near the target site under fluoroscopy using the position of the ribs as a guide; however, when confirmed by CT imaging, it shifted in the anteroposterior direction. A representative case is shown in Figure S2 in the Supporting Information. In general, the VBN can navigate the bronchus up to the 6th generation [24] and to peripheral lesions in most cases. However, selecting the bronchi up to the 6th generation may not be possible using a thin bronchoscope. The median number of bronchi that could be reached with a thin bronchoscope in this study was the 5th generation. In cases where the last bronchus navigated by the VBN cannot be selected, it might be better to change from fluoroscopy to cone-beam CT.

Although these issues remain to be resolved, the outcomes of RFID tag placement with fluoroscopic bronchoscopy in combination with VBN guidance are clinically acceptable.

This study had several limitations. This study was conducted on a limited number of cases at a single institution. In addition, there was selection bias because cases in which RFID tag placement was considered difficult were excluded. Large-scale studies from multiple institutions are required to ensure the accuracy of RFID tag placement under fluoroscopic bronchoscopy. Second, the accuracy of RFID tag placement is undeniably influenced by the bronchoscopist’s technique. However, we believe that the use of a VBN system will help ensure a certain level of quality.

We demonstrated that in over 80% of the cases, RFID tags can be placed near the target placement site using fluoroscopic bronchoscopy when combined with VBN guidance. RFID tag markings were found to be safe and made a significant contribution to segmentectomy. This outcome is clinically acceptable, and we believe that our RFID tag placement method using fluoroscopic bronchoscopy combined with VBN guidance will become more widespread in the future.

### Supplementary Information

Below is the link to the electronic supplementary material.

Supplementary file1 (DOCX 1660 kb)

A virtual bronchoscopic navigation image. Supplementary file2 (MP4 28057 kb)

Bronchoscopic procedure for RFID tag placement. Supplementary file3 (MP4 37940 kb)

Flow from preoperative consideration to actual RFID tag placement: right S7 segmentectomy. Supplementary file4 (MP4 35518 kb)

## Data Availability

The datasets generated and/or analysed during the current study are not publicly available but are available from the corresponding author upon reasonable request.

## Abbreviations

CT: Computed tomography
ENB: Electromagnetic navigation bronchoscopy
GGN: Ground-glass nodule
RFID: Radiofrequency identification
VAL-MAP: Virtual-assisted lung mapping
VBN: Virtual bronchoscopic navigation
