# Supplementary material for: Evaluation of radiofrequency identification tag accuracy using bronchoscopy with fluoroscopy and virtual navigation guidance before segmentectomy
Source: Surg Endosc. 2024 Aug 1;38(9):5438–45. doi: 10.1007/s00464-024-11110-4 (PMC11362373; doi:10.1007/s00464-024-11110-4)
Supplement: Supplementary file 1 — Supplementary file1 (DOCX 1660 kb) [file 464_2024_11110_MOESM1_ESM.docx]

# **TITLE:**

**Evaluation of preoperative RFID tag accuracy using fluoroscopic bronchoscopy with VBN**

# **Manuscript ID:** SEND-D-24-00899

**Authors’ full names:**

Masamichi Komatsu^1^, Kentaro Miura^2^, Miwa Yamanaka^1^, Yusuke Suzuki^1^, Taisuke Araki^1^, Norihiko Goto^1^; Jumpei Akahane^1^; Kei Sonehara^1^, Shunichiro Matsuoka^2^, Takashi Eguchi^2^, Kazutoshi Hamanaka^2^, Kimihiro Shimizu^2^, Masanori Yasuo^1,3^ and Masayuki Hanaoka^1^

**Authors’ affiliation(s):**

^1^First Department of Internal Medicine; ^2^Division of General Thoracic Surgery, Department of Surgery, Shinshu University School of Medicine, Matsumoto, Japan

^3^Department of Clinical Laboratory Sciences, Shinshu University School of Health Science, Matsumoto, Japan

**Figure S1: Radiograph of the surgical specimen**

**
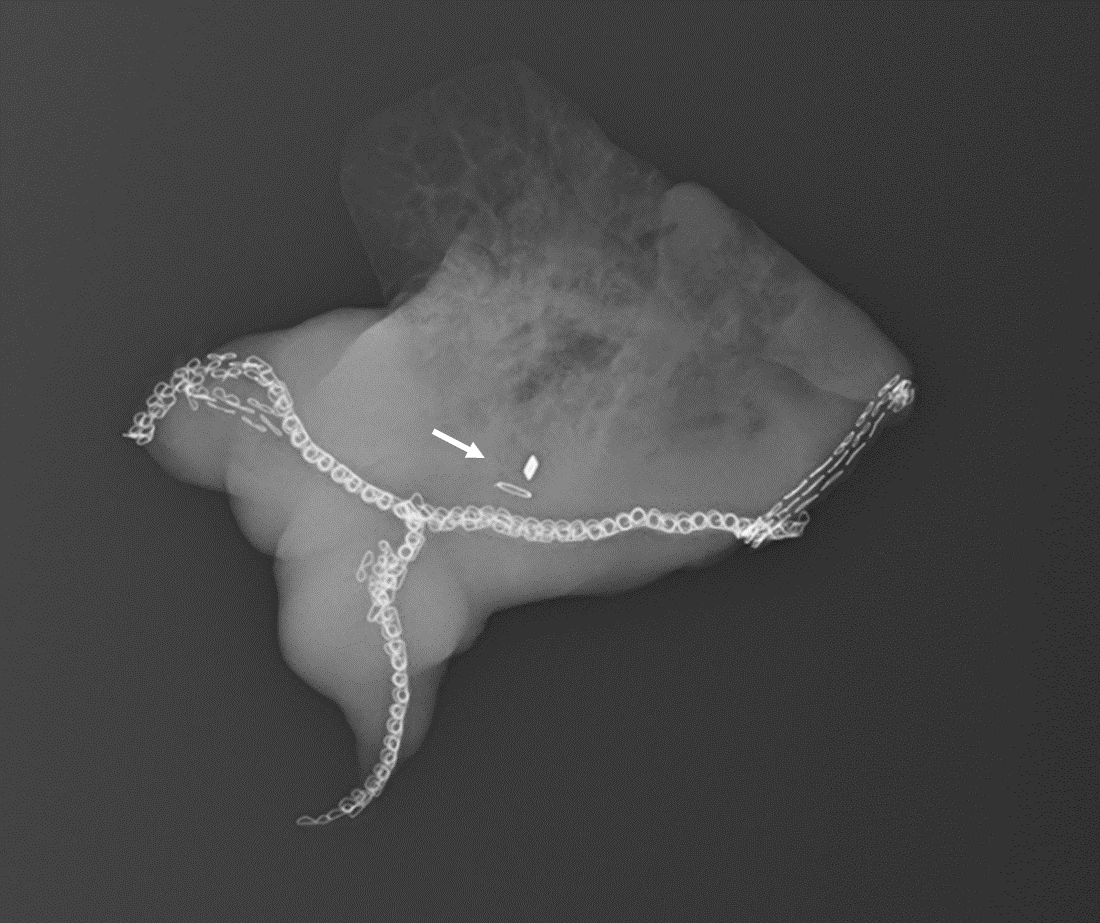
**

Radiograph of the resected specimen in the case shown in Fig 2. Radiograph showing the RFID tag (arrow) inside the resected specimen.

RFID: radiofrequency identification.

**Figure S2: Representative images in the case with a ‘failed’ RFID tag placement**

**
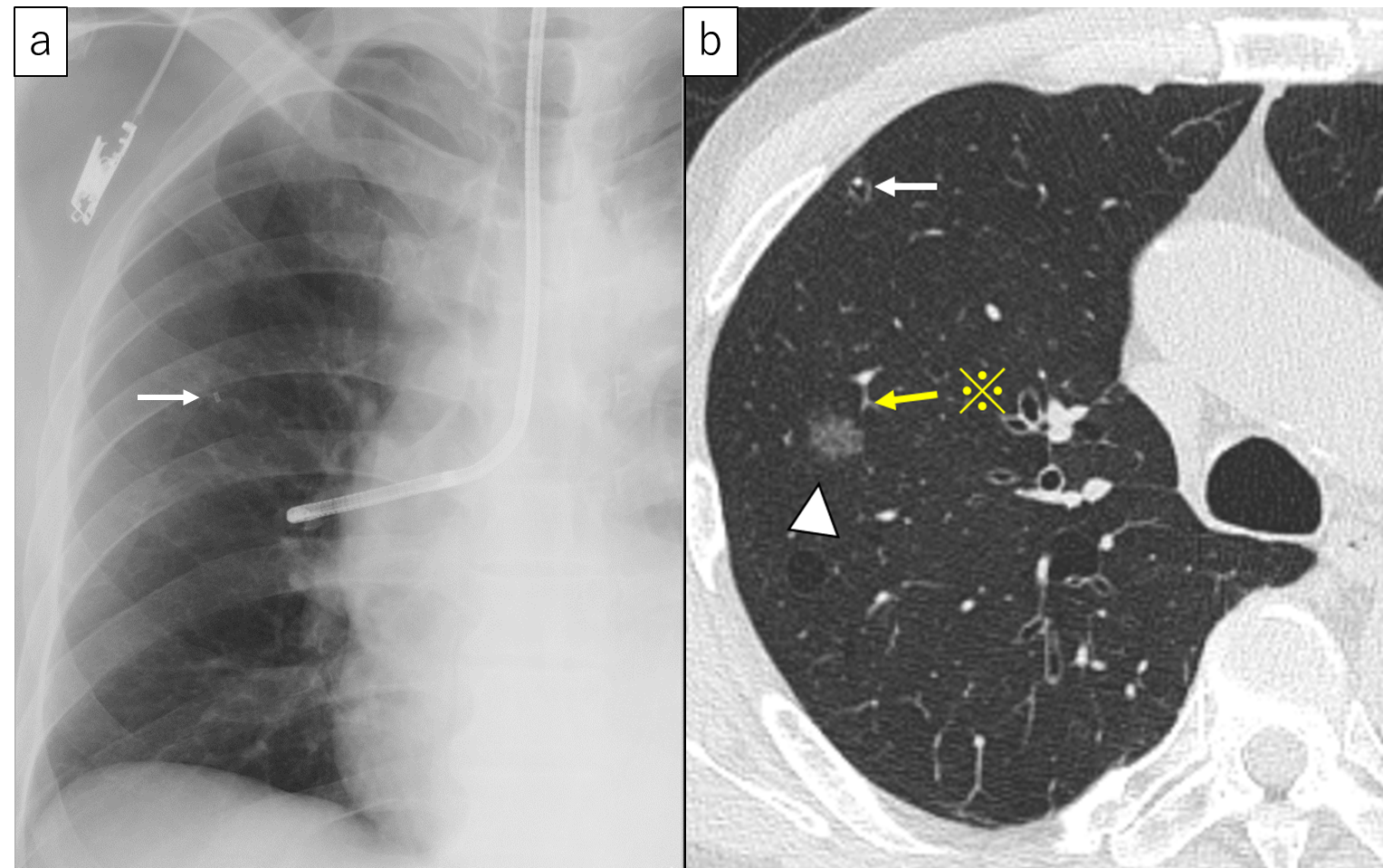
**

The ventral side of the tumour (arrowhead) is set as the target placement site (asterisk, arrow in yellow). (a) We selected the bronchus with reference to VBN, but it was difficult to select the final bronchus. Therefore, under fluoroscopy, an RFID tag (white arrow) was placed near the tumour using the position of the ribs as a guide. (b) CT scan after bronchoscopy revealed that the actual RFID tag (arrow, in white) location had shifted in the anteroposterior direction, which was judged to be ‘failed.’

RFID, radiofrequency identification.

**Table S1. Relationship between tumour appearance on CT and contribution to the surgery**

|  |  | **Tumour appearance on CT** | | |
| --- | --- | --- | --- | --- |
|  |  | **Pure GGN** | **Part-solid nodule** | **Solid nodule** |
| **Contribution to surgery** | **Necessary** | 10 | 2 | 2 |
|  | **Useful** | 5 | 6 | 3 |
|  | **Unnecessary** | 1 | 0 | 0 |

CT, computed tomography; RFID, radiofrequency identification.
